# Music Therapy Supports Children with Neurological Diseases during Physical Therapy Interventions

**DOI:** 10.3390/ijerph19031492

**Published:** 2022-01-28

**Authors:** Susann Kobus, Franziska Bologna, Ines Maucher, Daniel Gruenen, Ramona Brandt, Martin Dercks, Otfried Debus, Eva Jouini

**Affiliations:** 1 Clinic for Pediatrics I, Essen University Hospital, University of Duisburg-Essen, 45147 Essen, Germany; 2 Neurologic Clinic for Acute Neurology and Stroke Unit-Segeberger Klink, 23795 Bad Segeberg, Germany; franziska.bologna@segebergerkliniken.de; 3 Department of Pediatrics-Neuropaediatrics, Clemenshospital, 48153 Munster, Germany; i.maucher@alexianer.de (I.M.); d.gruenen@alexianer.de (D.G.); r.brandt@alexianer.de (R.B.); m.dercks@alexianer.de (M.D.); o.debus@alexianer.de (O.D.); e.jouini@alexianer.de (E.J.)

**Keywords:** music therapy, neurological diseases, pediatrics, physical therapy, hospitalized children, neurological early rehabilitation

## Abstract

Recent research found evidence supporting music therapy for children with neurological diseases during their hospitalized neurological early rehabilitation to promote their development during physical therapy. We hypothesized that live music therapy might improve vital signs during a physical therapy session. Seventeen children received live music therapy during the physical therapy session twice a week. Two more physical therapy sessions per week were held without music therapy. Heart rate, respiratory rate and oxygen saturation were recorded from 15 min before to 15 min after the therapy sessions. Physical therapy interventions showed changes in heart rate, respiratory rate and oxygen saturation between, before and after the sessions with or without music therapy. Live music therapy was effective for the vital signs during the intervention. We observed significantly lower heart and respiratory rates and higher oxygen saturation during physical therapy intervention with live music therapy in general (mean differences −8.0 beats per min; −0.8 breaths per min and +0.6%). When physical therapy was applied without music therapy children’s heart rates increased by 8.5 beats per min and respiratory rates increased by 1.0 breaths per min. Live music therapy leads to a decrease in heart and respiratory rates and an increase in oxygen saturation in children with neurological diseases during physical therapy with live music therapy. Music therapy supports the children in physical therapy interventions during their hospitalization.

## 1. Introduction

Patients with acute brain injuries from, for example, car accidents, falls and tumors can experience long-term health problems. After the brain injuries, the patients suffer from many restrictions, such as in mobility, language, motor skills, cognition and perception [1,2,3,4]. The treatment in a neurological early rehabilitation unit includes various forms of therapy in addition to medical care, such as occupational therapy, physical therapy, speech therapy and art therapies such as music therapy.

Music therapy is an established health profession in which music is used within a therapeutic relationship to address the physical, emotional, cognitive and social needs of individuals [5]. Previous studies have shown that music therapy has a stabilizing and relaxing effect on preterm infants and their parents, improving the infants’ general behavioral status and vital signs, such as oxygen saturation, heart rate and respiratory rate [6,7,8,9,10]. Several studies with various neurological diseases in adults have shown that music therapy also has a stabilizing and supportive effect on patients in early rehabilitation. According to a study with stroke patients, regular self-directed listening to music during the early phase after a stroke improves cognitive recovery and prevents negative mood [11]. Brain imaging studies have shown that music has strong connections to attention and memory systems [12]. In addition, music stimulation has also improved performance in tests of patients with autobiographical memory in dementia patients and in tests of visual neglect in stroke patients [13]. A further study of hospitalized stroke patients in early rehabilitation within two weeks after the stroke showed improved mood and increased flexion of the shoulder and elbow joint during music-movement therapy. The results of this study suggest that stroke rehabilitation should begin as early as possible, including during hospitalization. Nursing practice should include the concept of combining music and movement in order to improve the physical and psychological condition of stroke patients from the acute phase [14].

The aim of our study is to draw attention to the use of live music therapy and its elements to support various therapeutic methods, particular in physical therapy. In a study performed by Horne-Thompson and Barney, researchers found that using music therapy as a part of an interdisciplinary practice involving physical therapy improves functional outcomes [15].

Although there is an increase in the amount of research being published about the effectiveness of music therapy, but there is still a lack of research involving a combination of music therapy and physical therapy.

The aim of this study was to examine the effects of live music therapy in physical therapy on children’s vital signs heart rate, respiratory rate and oxygen saturation.

We hypothesized that music therapy stabilizes the vital signs of hospitalized children with neurological diseases during their physiotherapeutic intervention in neurological early rehabilitation to support the performance of physical therapy. In children with neurological diseases, the efficacy of music therapy during physical therapy has not been studied before.

## 2. Methods

### 2.1. Study Design

The study was designed as a prospective clinical trial. Apart from music therapy, there were no differences in medical care. For this paper, we analyzed the monitoring data of physical therapy sessions with music therapy and physical therapy sessions without music therapy from 17 patients at Phase B rehabilitation at the Clemenshospital Muenster.

### 2.2. Eligibility and Recruitment

After conclusion of emergency care for severe neurological diseases patients in Germany are admitted at an early stage to so-called Phase B rehabilitation. Children who were hospitalized at the Phase B rehabilitation at the Clemenshospital Muenster between September 2020 and June 2021 were eligible for the study. The target ages for recruitment ranged from 0 to 18 years. There were no inclusion or exclusion criteria for any particular neurological disease. All children who were treated in the described ward during the study period could not be excluded from the study because of their illness. Exclusion criteria were that the children were not connected to the monitor during the physical therapy sessions or were in a palliative care. Parental informed consent was obtained during the first week of hospitalization.

### 2.3. Intervention

Physical therapy and music therapy were carried out for each child individually. Vital signs data heart rate, respiratory rate and oxygen saturation were saved on the monitor 15 min before, during and 15 min after each therapy session. Vigilance and individual characteristics of the child were documented in a protocol. Clinical data were retrieved from the patients’ medical record. The physical therapy was carried out according to the same program, regardless of whether music therapy was included or not.

#### 2.3.1. Physical Therapy

During the hospitalized neurological early rehabilitation, the therapy interventions were designed to help the patient achieve functional goals. Interventions included exercises to improve the patient’s mobility.

Physical therapy was performed between four and six times a week in clinically stable patients starting directly after admission to the hospital. The timing of each therapy session was coordinated by the physical therapist, nursing staff and parents.

The physical therapy interventions were compiled with the neurological treatment techniques and concepts of Vojta Therapy and the Bobath concept and elements of respiratory therapy.

Vojta therapy is used as a basic therapy for movement disorders and numerous diseases. The reflex locomotion is triggered mainly from the three basic positions of the prone, back and side position and from ten trigger zones on the body described by Vojta. In healthy newborns, the complete activation of the movement pattern “reflex crawling” is possible from a single zone. In older children and adults, several zones have to be stimulated to trigger reflex locomotion. In precisely defined starting positions, the Vojta therapist exerts targeted pressure on certain areas of the patient’s body. Irrespective of the patient’s will, this stimulus leads to two movement complexes in people of all ages, which contain all the essential needs of human movement and straightening: “reflex crawling” and “reflex turning”.

The automatic and unconscious access to the elements of the erection and locomotion—grasping and handling, turning around and getting up, walking and running—is a matter of course for healthy people. However, if the central nervous system and the postural and musculoskeletal system are damaged, this is no longer usable. Vojta therapy stimulates ten trigger zones on the body in three basic positions (prone, back and side position). This so-called reflex locomotion makes movement patterns accessible again in parts. In children’s therapy, the natural urge to move, curiosity and the joy of movement to offer the child new opportunities and perspectives are used. The physical therapist has the opportunity to develop a relationship with a patient and to influence both, attitudes and behavior. It is important that the patient experiences self-efficacy and the pain complaints of the patient are the focus. The aim is to strengthen and promote the child’s independence. Bobath Therapy takes the person in his holistic personality and treats them accordingly. Through targeted grips and postures, movement sequences are positively influenced, and new movement strategies are developed.

#### 2.3.2. Music Therapy

Music therapy was performed during the physical therapy sessions twice weekly in clinically stable patients starting from the second week of hospitalization. The timing of each therapy session was coordinated by the physical therapist, music therapist, nursing staff and parents.

Each session consisted of individual playing of a few musical notes of the instrument sansula by the music therapist. Depending on the infant’s condition, the improvised playing was adapted to the children’s breathing and reactions, beginning with humming tones that were followed by tone sequences. The tempo was adjusted according to breathing and heart rates. The sounds were adapted to the physical therapy intervention. During breaks in therapy, deep and slow tones were played in order to create a calm atmosphere. During difficult exercises of physical therapy, where a lot of strength was required by the child, the music therapist played a little bit faster and the melody moved upwards. When it became easier again for the child and less exertion was required, the melody also moved back to lower tones. The music supported the exercises of physical therapy and created a calm atmosphere between the exercises.

The sansula consists of a wooden ring covered with an eardrum, on which a small kalimba is attached. It creates a space-filling, long-lasting and soft sound. This way of performing music therapy has already been shown to be a very effective method in several studies with preterm infants and their parents. The sound of the Sansula improved the vital signs heart rate, respiratory rate and oxygen saturation in the premature infants, and the parents described a relaxing effect of the sounds [6,16].

The strongest argument for including music therapy in neurorehabilitation lies in its neurological benefits. Neurologically, music is intrinsically rewarding as it activates brain regions involved in reward, motivation, emotion and arousal [17]. Cortical changes in brain damaged patients during music interventions indicate activation of bilateral networks across the frontal, temporal and parietal lobes, cerebellum and limbic areas, stimulating cognitive, motor and emotional processes [11,18,19]. When integrated with repetitive rehabilitation exercises and drills, music therapy that is tailored to an individual’s performance can enhance the motivation to sustain engagement and may improve patient mood and enhance motivation [20].

### 2.4. Statistical Analysis

Quantitative variables are presented as mean and 95% confidence (Cl) intervals or standard deviation, respectively; for qualitative factors, absolute and relative frequencies are given. Vital signs were stratified into heart rate, respiratory rate and oxygen saturation. To compare the values of vital signs before, during and after music therapy, paired t-tests were performed.

All statistical calculations were performed using IBM SPSS Statistics 27. (IBM, Chicago, IL, USA) *p*-values < 0.05 were considered significant.

## 3. Results

### 3.1. Patients

Seventeen children with neurological diseases were included into the study at the Phase B rehabilitation at Clemenshospital Münster between September 2020 and June 2021. Seven children were excluded. The reasons for exclusion were that the children had not been connected to the monitor during the physical therapy sessions or were in a palliative care. All families who were asked to take part in the study agreed for participation.

The children in the study had acquired brain injuries from accidents (24%), viral infections (12%), brain attacks due to previous illnesses (29%) or birth as preterm infant or ill newborn (35%). They had a mean age of 38 months (range 1 to 134 months) (Table 1). Table 1 shows the clinical characteristics of the included patients.

### 3.2. Therapy Sessions

A total of 256 physical therapy sessions were conducted, 128 with music therapy and 128 without music therapy. Each child got the same number of each kind of session. The mean duration of each therapy session was 44 min (range 21 and 71 min).

### 3.3. Analyses of Vital Signs during Physical Therapy Sessions with and without Music Therapy

#### 3.3.1. Heart Rate

Baseline heart rate before physical therapy session with music therapy decreased during the session. We found a decrease during therapy of 8.0 beats/min (95% CI 6.6–9.5). After physical therapy session with music therapy, we found a slight increase in heart rate of 1.5 beats/min, but we saw an overall decrease from before to after therapy of 6.5 beats/min (95% CI 4.5–8.6). Without music therapy, we saw an increase in heart rate during therapy of 8.5 beats/min (95% CI 6.7–10.3) and a decrease after therapy of 3.4 beats/min. Overall, we found an increase in heart rate from before to after physical therapy without music therapy of 5.0 beats/min (95% CI 2.6–7.5) (Table 2 and Table 3).

#### 3.3.2. Respiratory Rate

We saw a decrease of 0.8 breaths/min (95% CI 0.1–1.5) in the respiratory rate during the intervention with music therapy compared to the respiratory rate before the intervention (Table 2 and Table 3). After the physical therapy session with music therapy, we had an additional decrease of 0.1 breaths/min. We saw an increase of 1.0 breaths/min (95% CI 0.3–1.8) in the respiratory rate during the physical therapy session without music therapy and an additional increase in the respiratory rate of 0.1 beats/min.

#### 3.3.3. Oxygen Saturation

We found an increasing oxygen saturation during the physical therapy with music therapy of 0.6% (95% CI 0.2–1.1). After the session, we saw an additional increase of 0.2%). When comparing the oxygen saturation before and after the physical therapy session with music therapy, we found an increase of 0.8% (95% CI 0.3–1.4). In physical therapy without music therapy, we found a slight decrease in oxygen saturation between before and during the session of 0.1% and an additional slight decrease between during and after the therapy session of 0.2% which was not significant (*p* = 0.581) (Table 2 and Table 3).

### 3.4. Analyses of the Therapeutic Courses with and without Music Therapy

Out of 256 sessions, 128 (50%) were performed with and 128 (50%) without music therapy.

#### 3.4.1. Heart Rate

**Figure 1:**
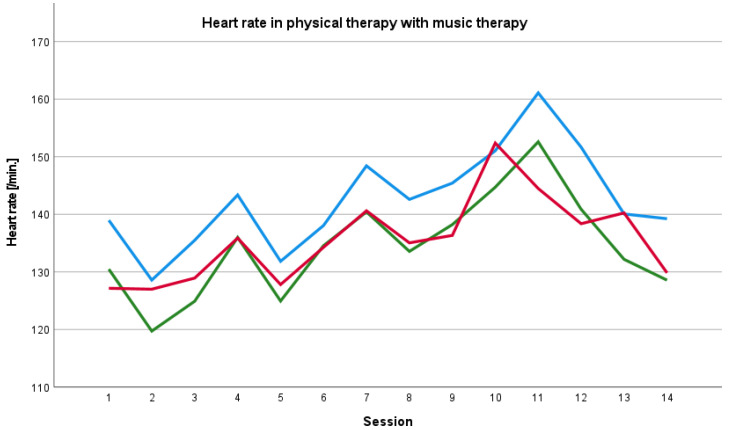
Baseline, peri- and post-therapy heart rate in physical therapy with music therapy. Blue line: mean baseline value, green line: mean peri-therapy value, red line: mean post-therapy value.

**Figure 2:**
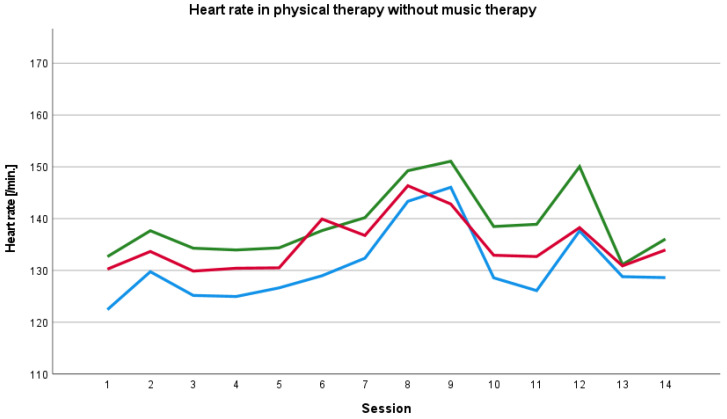
Baseline, peri- and post-therapy heart rate in physical therapy without music therapy. Blue line: mean baseline value, green line: mean peri-therapy value, red line: mean post-therapy value.

The baseline heart rate before the physical therapy session with music therapy was constantly higher than the heart rate during and after the session and the baseline heart rate before physical therapy session without music therapy was constantly lower the than heart rate during and after the session. We found an increase in the heart rate during the course of therapy until the 11th session and a decrease after the 11th session (Figure 1). We also saw an increase in the heart rate during the course of therapy without music therapy until the 9th session (Figure 2).

#### 3.4.2. Respiratory Rate

**Figure 3:**
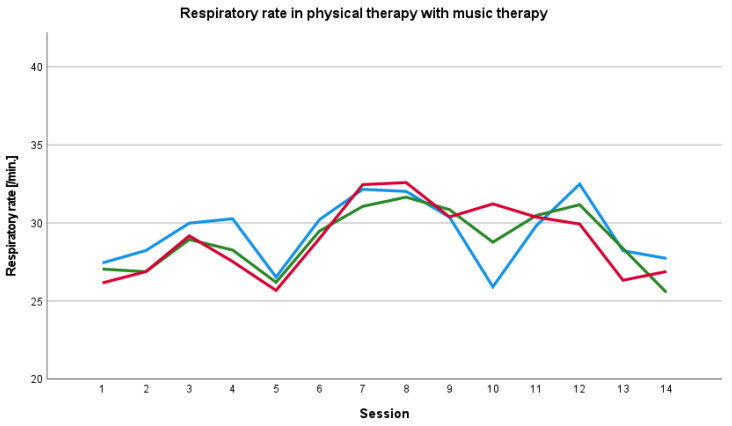
Baseline, peri- and post-therapy respiratory rate in physical therapy with music therapy. Blue line: mean baseline value, green line: mean peri-therapy value, red line: mean post-therapy value.

**Figure 4:**
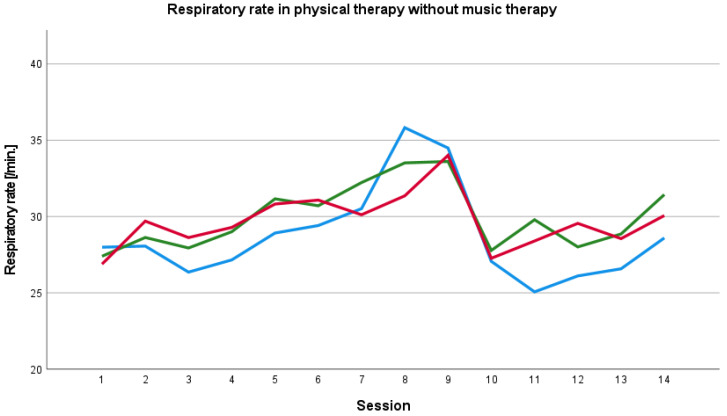
Baseline, peri- and post-therapy respiratory rate in physical therapy without music therapy. Blue line: mean baseline value, green line: mean peri-therapy value, red line: mean post-therapy value.

The baseline respiratory rate before physical therapy session with music therapy was overall higher than the respiratory rate during and after the session. We also found an increase in the respiratory rate during the course of therapy in the 8th session (Figure 3; Figure 4).

#### 3.4.3. Oxygen Saturation

**Figure 5:**
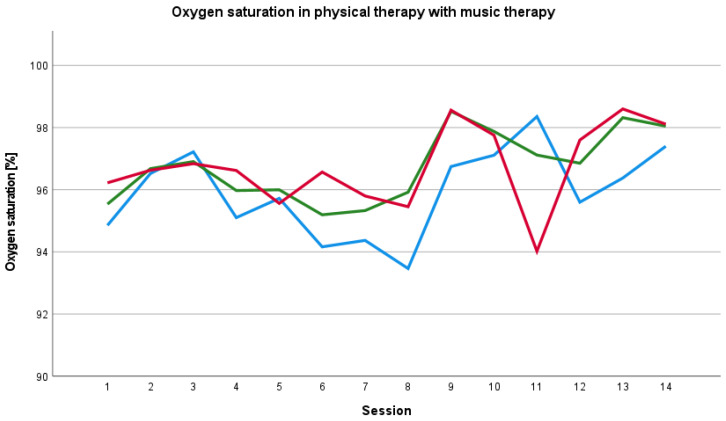
Baseline, peri- and post-therapy oxygen saturation in physical therapy with music therapy. Blue line: mean baseline value, green line: mean peri-therapy value, red line: mean post-therapy value.

**Figure 6:**
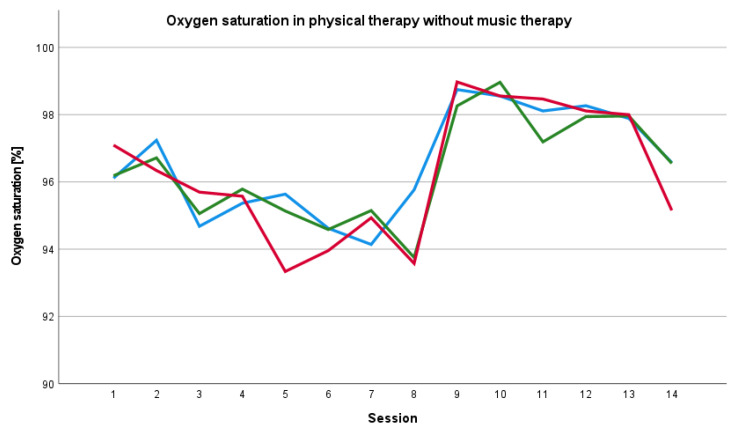
Baseline, peri- and post-therapy oxygen saturation in physical therapy without music therapy. Blue line: mean baseline value, green line: mean peri-therapy value, red line: mean post-therapy value.

In most physical therapies with music therapy, the baseline oxygen saturation before session with music therapy was lower than the oxygen saturation during and after the session (Figure 5). We saw a changing relationship between the oxygen saturation values before, during and after physical therapy without music therapy (Figure 6).

## 4. Discussion

In this prospective intervention study with live music therapy during a physical therapy intervention, it was found to be that music therapy stabilizes vital signs in children with neurological diseases during and after a therapy, reflected by decreased heart and respiratory rates and increased oxygen saturation during and after sessions. We found significant decreases in the heart rate (*p* = 0.000) and respiratory rate (*p* = 0.025) and significant increases in the oxygen saturation (*p* = 0.005) in physiotherapeutic interventions with music therapy.

The general changes in vital signs were in line with previously published data. Several studies showed that music therapy had a stabilizing and relaxing effect on the preterm infant’s general behavioral state, sleep patterns and vital signs. Music therapy has also beneficial effects on vital signs when delivered to sleeping preterm infants [6]. A transient stabilization of vital signs is not the only purpose of interventions in the hospitalized neurological early rehabilitation, but improving the vital signs in therapeutic interventions can have a long-term effect on the modulation of the child’s stress level. Several studies have investigated that music can help in many aspects of the brain, including pain reduction, stress relief, memory and brain injuries [21].

Our study adds new evidence that music therapy is also effective during physical therapy interventions, which shows the importance for an interdisciplinary cooperation between several forms of therapy.

Despite our results, the study also presents limits. Due to the significantly improved vital signs during physical therapy, which were accompanied by music therapy, the children may achieve their physical therapy goals faster. Further studies should investigate this question and subdivide a larger group of patients into the various children’s conditions. In our study, music therapy sessions were performed at different times of the day, dependent on the clinical examinations. A future study should investigate whether music therapy can also support the other forms of therapy, such as occupational therapy, speech therapy and sports therapy. Additional prospective studies are needed to explore if music therapy can also be effective in physical therapy interventions for patients with other diseases and other age groups to expand the interdisciplinary collaboration in hospitals.

## Figures and Tables

**Table 1:** Clinical characteristics of the participants.

	Patients (*n* = 17)
Male gender, *n* (%)	14 (82%)
Age (month), mean (range)	38.2 (1–134)
Physical therapy sessions (with music therapy, without music therapy), *n*	256 (128, 128)
PEG/PEJ feeding, *n* (%)	
at admission	14 (82%)
at discharge	11 (65%)
Condition requiring intensive medical care, *n* (%)	
at admission	12 (71%)
at discharge	9 (53%)
Tracheostoma requiring suction, *n* (%)	
at admission	5 (29%)
at discharge	5 (29%)
Intermittent ventilation, *n* (%)	
at admission	4 (24%)
at discharge	3 (18%)
Disorientation, *n* (%)	
at admission	5 (29%)
at discharge	2 (12%)
Behavioral disorder (with danger to oneself and/or others), *n* (%)	
at admission	2 (12%)
at discharge	0
Severe communication disorder, *n* (%)	
at admission	17 (100%)
at discharge	15 (88%)
Swallowing disorder, *n* (%)	
at admission	14 (82%)
at discharge	13 (76%)

**Table 2:** Heart rate, respiratory rate and oxygen saturation before and during physical therapy sessions with and without music therapy.

Vital Sign	Music Therapy	*N*	Mean before Therapy (95% CI)	Mean during Therapy (95% CI)	Mean Difference (95% CI)	*p* Value
Heart rate [beats per min]	yes	128	139.0 (134.9–143.2)	131.0 (126.9–135.1)	−8.0 (−9.5−(−6.6))	0.000
	no	128	128.6 (124.5–132.8)	137.1 (133.1–141.1)	8.5 (6.7−10.3)	0.000
Respiratory rate [breaths per min]	yes	128	29.2 (27.8–30.7)	28.4 (27.2–29.7)	−0.8 (−1.5−(−0.1))	0.020
	no	128	28.5 (27.1–29.9)	29.5 (28.1–30.9)	1.0 (0.3−1.8)	0.009
SaO_2_ [%]	yes	128	95.7 (94.6–96.8)	96.3 (95.3–97.3)	0.6 (0.2−1.1)	0.004
	no	128	96.0 (94.9–97.0)	95.9 (94.8–97.0)	−0.1 (−0.7−0.5)	0.806

CI = confidence interval, SaO_2_ = oxygen saturation.

**Table 3:** Heart rate, respiratory rate and oxygen saturation before and after physical therapy sessions with and without music therapy.

Vital Sign	Music Therapy	*N*	Mean before Therapy (95% CI)	Mean after Therapy (95% CI)	Mean Difference (95% CI)	*p* Value
Heart rate [beats per min]	yes	128	139.0 (134.9–143.2)	132.5 (128.3–136.7)	−6.5 (−8.6−(−4.5))	0.000
	no	128	128.6 (124.5–132.8)	133.7 (129.6–137.7)	5.0 (2.6−7.5)	0.000
Respiratory rate [breaths per min]	yes	128	29.2 (27.8–30.7)	28.3 (26.9–29.6)	−0.9 (−1.8−(−0.1)	0.025
	no	128	28.5 (27.1–29.9)	29.6 (28.0–31.2)	1.1 (0.1−2.2)	0.040
SaO_2_ [%]	yes	128	95.7 (94.6–96.8)	96.5 (95.5–97.5)	0.8 (0.3−1.4)	0.005
	no	128	96.0 (94.9–97.0)	95.7 (94.6–96.9)	−0.3 (−0.9−0.5)	0.581

CI = confidence interval, SaO_2_ = oxygen saturation.

## Data Availability

Original data will be made available to any qualified researcher upon request.
